# Endothelial Dysfunction in Children With Nephrotic Syndrome: A Cross-Sectional Study

**DOI:** 10.7759/cureus.53628

**Published:** 2024-02-05

**Authors:** Harapriya Das, Amit Satapathy, Joseph John, Manisha Kar, Sudipta Mohakud

**Affiliations:** 1 Pediatrics, All India Institute of Medical Sciences, Bhubaneswar, IND; 2 Physiology, All India Institute of Medical Sciences, Bhubaneswar, IND; 3 Radiology, All India Institute of Medical Sciences, Bhubaneswar, IND

**Keywords:** fmd, cimt, pulse wave velocity, reactive hyperemic index, arterial stiffness, endothelial dysfunction, ns

## Abstract

Background

Children with nephrotic syndrome (NS) have a higher risk of cardiovascular morbidity. Studies on the evaluation of arterial stiffness and endothelial function and its predictive risk factors in these children are limited.

Objective

The primary objective of the study was to determine arterial stiffness by measuring carotid intimal medial thickness, flow-mediated dilatation, and physiological parameters in children with nephrotic syndrome to predict the risk of premature atherosclerosis as compared to controls.

Participants

A total number of 33 children with NS in the age group of 2-14 years in remission and 39 healthy controls were enrolled in the study. Out of 33 children with nephrotic syndrome, five were infrequently relapsing NS, eight were frequently relapsing, 16 were steroid dependent, and four were steroid-resistant NS.

Intervention

Relevant history, physical examination, anthropometric measurements, and laboratory investigations were done. Carotid intimal medial thickness (cIMT), flow-mediated dilatation (FMD), and other physiological parameters were measured in both children with NS and control groups.

Outcome

Carotid intimal medial thickness (cIMT), flow-mediated dilatation (FMD), and other physiological parameters were compared between children with NS and healthy controls for detecting arterial stiffness and endothelial dysfunction.

Results

Dyslipidaemia was seen in more than 50% of children during remission. There was neither significant difference in mean cIMT in the common carotid artery nor FMD between the control and study groups. There was a trend of lower Reactive Hyperemia Index (RHI) in children with NS.

Conclusion

Dyslipidemia persists even during the remission phase in NS. No statistically significant difference is observed in cIMT and percentage proportionate change in FMD in both the study and control groups. Nevertheless, RHI is notably lower in children with NS. These findings need further validation in future studies.

## Introduction

Nephrotic syndrome (NS) is the most common glomerulopathy of childhood, usually presenting between the age group of two to six years. It is characterized by nephrotic-range proteinuria, defined as proteinuria of more than 1gm/m2 daily, 40mg/m2/hr with hypoalbuminemia (≤3 g/dL), and edema [1]. The prevalence of nephrotic syndrome is 12-16 cases per 100,000 paediatric population (2). The incidence of NS in India is 9-10/100,000, which is higher than that of Western countries, which is 2-3/100000 [1]. Corticosteroids are the mainstay of therapy in NS. Usually, 80% of children respond to steroids and achieve remission after initial therapy of six weeks daily, followed by six weeks of alternate-day medication (2). However, relapse of proteinuria is an issue despite adequate treatment of the initial episode. Depending on the frequency of relapse, it has been classified as infrequently relapsing nephrotic syndrome (IFRNS), frequently relapsing nephrotic syndrome (FRNS), and steroid-dependent nephrotic syndrome (SDNS). Rarely, children may not respond to steroids despite adequate therapy, which is labeled as steroid-resistant nephrotic syndrome (SRNS) [2].

Children with SRNS progress towards chronic kidney disease (CKD), which is known to increase cardiovascular mortality 500 to 1000 fold compared to the general population in young adults [3]. Premature atherosclerosis is one of the inevitable complications of NS. Sustained hypoproteinemia, dyslipidemia, oxidative stress due to repeated infections, and steroid therapy are the main risk factors making these children more susceptible to premature atherosclerosis [4]. Additional risk factors, such as hypertension, obesity, insulin resistance, chronic inflammation, long-term treatment with steroids and other immunosuppressive drugs, such as cyclosporine A, hypercoagulability, and oxidative stress. Nephrotic range proteinuria is associated with a five to six-fold increased risk of myocardial infarction [5]. Due to their lifelong exposure to atherogenic risk factors, children and adolescents with idiopathic SRNS are at particularly high risk for developing premature atherosclerosis, so addressing these risk factors would prevent many cardiovascular complications. There are several studies regarding the association of biomarkers with atherosclerosis in NS, which are not reliable in predicting the severity of risk [6]. Several non-invasive methods have been used to assess premature atherosclerosis and endothelial dysfunction in adults, such as carotid intima-media thickness (IMT), pulse wave velocity (PWV) reflects arterial elasticity, flow-mediated dilatation (FMD), reactive hyperemia index and augmentation index [7,8]. However, studies on the evaluation of endothelial function and its predictive risk factors in children with NS are limited. There is a paucity of evidence regarding these parameters in determining endothelial dysfunction. Such studies are important from a therapeutic point of view as well as for planning preventive strategies. The primary objective of the study was to determine the arterial stiffness by measuring Carotid intimal medial thickness, flow-mediated dilatation, and physiological parameters in IFRNS, FRNS, SDNS, and SRNS children for predicting the risk of premature atherosclerosis as compared to controls.

## Materials and methods

This study was a cross-sectional study conducted during the period January 2020 to December 2021. Children with Idiopathic nephrotic syndrome (SSNS/ SDNS/ FRNS / IFRNS) of age group 2-14 years who were in remission and attending pediatric outpatient department (OPD) during the study period were included in the study. Children with a family history of premature atherosclerosis or familial hyperlipidemia (FH), estimated glomerular filtration rate (eGFR) <30 ml/min/1.73m², and those taking lipid-lowering agents were excluded from the study. Healthy children with BMI <85th centile and without dyslipidemia were taken as controls from OPD.

Ethical clearance was taken from the Institutional Ethics Committee of All India Institution of Medical Sciences, Bhubaneswar (IEC) before the initiation of the study. Children who came to the pediatric OPD or were admitted to the pediatric ward fulfilling the study inclusion criteria were enrolled after obtaining consent and assent (wherever applicable). They were given detailed information regarding the study in simple, understandable, local language. Demographic information, age of onset of NS, medication history (regarding the duration and type of steroids and any other alternative therapy like immunosuppressants), detailed course of illness, and family history of obesity, diabetes, hypertension, or hyperlipidemia in first-order relatives were recorded in a pre-designed proforma. Blood pressure was measured using the Spg-06 Aneroid sphygmomanometer (Morepen Laboratories Limited, Baddi, India), a manual blood pressure (BP) instrument, using an appropriate size cuff in a relaxed position. Interpretation of BP recording was done based on the American Academy of Pediatrics (AAP) 2017 recommendation. [9] Weight and height were measured to the nearest 0.1 kg and 0.5 cm, respectively, using appropriate standard techniques, and the BMI was calculated using the formula as mentioned below and classified according to the Indian Academy of Pediatrics (IAP) growth charts. The following laboratory investigations were done for all the enrolled children, which included fasting lipid profile, serum urea, creatinine, albumin, and urine dipstick. Serum albumin, creatinine, and urea were analyzed by AU680 clinical chemistry analyzer (Beckman Coulter, Brea, California).

IU22 12-15 Hz images (Philips, Amsterdam, Netherlands) using high resolution with brightness mode (B-mode) ultrasound with linear probe was used for this estimation of carotid intimal medial thickness (cIMT). Measurements were obtained from the far wall from three segments (distal common carotid - 10 mm proximal to the carotid bulb, carotid bulb, and the proximal internal carotid) of both sides. The measurements were made on each side; the mean of left and right common carotid arteries were used in the study. The IMT from both sides was further averaged to give the overall mean IMT [10]. Flow-mediated dilatation of the brachial artery was measured by using a Doppler ultrasound [11]. The measurement of cIMT and FMD was done by an independent, experienced radiologist who is completely blinded.

Physiological parameters were recorded in the Clinical Physiology Laboratory in the Department of Physiology. The pulse waveforms were recorded by placing pulse transducers (Miller's pulse transducer; AD Instruments, Sydney, Australia) simultaneously on the carotid and radial arteries for five minutes. The PowerlabTM 4/35 hardware and LabchartTM 8 reader software were used to analyze the data (AD Instruments, Sydney, Australia). The sample acquisition frequency was set at 1000 Hz. The components below 50 Hz were stored using a low pass filter, and the wavefronts were determined. Carotid-radial pulse wave velocity (crPWV) was calculated as D/t [12]. Physiologically, endothelial dysfunction is screened by the detection of impaired flow-induced reactive hyperemia in the fingertip. Reactive hyperemia index (RHI) was calculated as the fold increase in pulse wave amplitude relative to baseline [13]. Peripheral BP, central BP, augmentation index (AIx%), and heart rate were recorded by the equipment (BP+ cardioscope II; USCOM Ltd., New South Wales, Australia). USCOM BP+® employs supra-systolic oscillometric technology to compute central blood pressure. Physiological parameters were performed and analysed by an experienced physiologist unaware of the status of the child.

Statistical analysis

The collected data were entered in Microsoft Excel (Microsoft, Redmond, Washington). The normality of the data was examined using the Kolmogorov-Smirnov test using SPSS version 20 (IBM Inc., Armonk, New York). Continuous data were presented as mean and standard deviation (SD), and categorical data were presented as frequencies and percentages (%). Comparison between mean values of the continuous variables was analyzed by student T-test for normally distributed data, whereas the Mann-Whitney U-test was used for those not normally distributed. Categorical variables were analyzed using the Chi-squared test and Fischer's exact text. Pearson's correlation was employed to assess the association between radiological and physiological parameters, used for the early detection of arterial stiffness, and a p<0.05 was considered to be statistically significant.

Sample size calculation

The sample size was calculated based on the mean value of cIMT in previously published research articles [14]. The mean value of cIMT in children with NS and in apparently healthy children of the age group of 2-14 years was reported to be 0.46±0.06 mm and 0.42±0.05 mm, respectively [15]. Considering a power of 80% and a confidence interval of 95%, the calculated sample size for the study was estimated to be 30 in each group. Assuming attrition to be 10%, we calculated the sample size to be 33 in each group. The sample size was calculated using online statistical software (Raosoft Inc., Seattle, Washington).

## Results

After taking into consideration the inclusion and exclusion criteria, 72 children were enrolled in this study. Thirty-three children were in the NS group, whereas the rest, 39, were controls. Among the 72 children, 42 were boys and 30 were girls. The mean age of the study group was 7.2 ± 2.8 years, and that of the control group was 9.9 ± 2.7 years. There was a significant difference between the mean values of height and weight of the study and control group. The mean BMI of the study group was 16.38 ± 2.58, and that of the control was 15.77 ± 2.63, with mean Z-scores of 0.251 ± 1.16 and -0.57 ± 1.02, respectively. The details of the demographic and anthropometric parameters have been mentioned in the table below (Table 1).

**Table 1:** Comparison of baseline physical and biochemical parameters between the study group and controls Normotriglyceridemia: TG < 100 (0–9 years), and <130 (10–19 years); Hypertriglyceridemia: Hypertriglyceridemia: TG ≥100 (0–9 years), and ≥130 (10–19 years) T test was used for continuous variables, Chi-square and Fisher exact for categorical variables. A p-value of <0.05 was considered significant TC - total cholesterol; HDL - high-density lipoprotein; LDL - low-density lipoprotein

Parameter	Study group (n=33) mean ± SD or number (%)	Control (n=39) mean ± SD or number (%)	p-value
Age (years)	7.21 ± 2.83	9.92 ± 2.76	<0.001
Gender			
Boys	23 (69%)	19 (49%)	0.072
Girls	10 (31%)	20 (51%)	
Height (cm)	115.5 ± 14.8	131.6 ± 14.4	0.0001
Height Z-Score	-0.853 ± 1.6	-0.623 ± 1.32	0.5
Weight (kg)	22.5 ± 9.0	22.67 ± 7.93	0.0128
Weight Z-score	-0.303 ± 1.45	-0.744 ± 1.13	0.15
BMI	16.38 ± 2.58	15.77 ± 2.63	0.33
BMI Z-score	0.251 ± 1.16	0.57 ± 1.02	0.002
Total cholesterol (mg/dl)	292.2 ± 127.7	139.4 ± 37.5	<0.001
TC (< 200mg/dl)	9 (27%)	37 (95%)	<0.001
TC (> 200mg/dl)	24 (73%)	2 (5%)	
Triglycerides (mg/dl)	200 ± 34	111 ± 36	<0.001
Normotriglyceridemia	7 (21%)	26 (67%)	<0.001
Hypertriglyceridemia	26 (79%)	13 (33%)	
HDL (mg/dl)	64 ± 21	41 ± 8	<0.001
>40 mg/dl	33 (100%)	19 (49%)	<0.001
<40 mg/dl	0	20 (51%)	
LDL (mg/dl)	159.84 ± 80.8	86.5 ± 27.8	<0.001
<130 mg/dl	18 (55%)	37 (95%)	<0.001
>130 mg/dl	15 (45%)	2 (5%)	
Serum albumin (gm/dL)	3.2 ± 1.5	4.17 ± 0.7	0.0013

Among the children with NS, the most common group was SDNS (48%), whereas only four children had SRNS. Almost 60% of these children were receiving immunosuppressants. A sizeable number of those were on mycophenolate mofetil (MMF) therapy (36%) as a steroid-sparing agent (Table 2).

**Table 2:** Baseline characteristics of children with NS NS - nephrotic syndrome; SDNS - steroid-dependent nephrotic syndrome; FRNS - Frequently relapsing nephrotic syndrome; IFRNS - infrequently relapsing nephrotic syndrome; SRNS - steroid-resistant nephrotic syndrome; MMF - mycophenolate mofetil

Parameters	Mean ± SD (n=33)
Age	7.21 ± 2.83 years
Gender	
Boys	23 (69.6%)
Girls	10 (30.4%)
Mean age of onset of disease (years)	4.4 ± 2.71
Duration of NS (months)	34.69 ± 24 (12-108) months
Total number of relapses	4 ± 2.27
Types of NS	
SDNS	16 (48%)
FRNS	8 (25%)
IFRNS	5 (15%)
SRNS	4 (12%)
Treatment	
Cyclosporine	5 (15%)
MMF	12 (33%)
Tacrolimus	1 (3%)
Levamisole	3 (9%)
Blood pressure	
Normotensive	21 (63.7%)
Elevated blood pressure	0 (0%)
Stage 1 hypertension	9 (27.2%)
Stage2 hypertension	3 (9.1%)

Dyslipidaemia was seen in more than 50% of children during remission. Hypertension was seen in almost 36% of children as per AAP 2017 recommendation. Hypercholesterolemia was documented in 73% of children, whereas hypertriglyceridemia and elevated low-density lipoprotein (LDL) were observed in 79% and 45% of children, respectively. We could not find any difference in baseline characteristics or biochemical profiles among the different subcategories of NS (Table 3).

**Table 3:** Comparison of physical, biochemical, and radiological parameters between subcategories of NS TG - triglyceride; LDL - low-density lipoprotein; HDL - high-density lipoprotein; cIMT - carotid intima medial thickness; FMD - flow-mediated dilatation; crPWV - carotid radial pulse wave velocity Cholesterol, TG, LDL, and HDL were presented as median with Interquartile range (IQR). Rest of the values are mean with standard deviation. Gender (nine boys and girls) were presented as proportion. ANOVA test was applied for comparison between the subgroups.

Parameters	IFRNS (n=5)	FRNS (n=8)	SDNS(n=16)	SRNS (n=4)	p-value
Age (years)	7.2 ± 3	6 ± 2.1	7.6 ± 3	7.8 ± 3.5	0.58
Gender					
Boys	3 (60%)	5 (62.5%)	13 (81.2%)	2 (50%)	0.57
Girls	2 (40%)	3 (37.5)	3 (18.8%)	2 (50%)	
Weight (kg)	17.4 ± 4.3	18 ± 4.2	26 ± 8.2	21 ± 8.5	0.02
Height (cm)	108 ± 12	106 ± 7.1	122 ± 15	112 ± 14	0.04
BMI (kg/m^2^)	14.6 ± 0.7	15.5 ± 2.02	17.2 ± 3.3	16.5 ± 4.8	0.17
Cholesterol (mg/dl)	148 (104-278)	289 (222-364)	223 (173-300)	352 (148-477)	0.88
TG (mg/dl) IQR	123 (71-230)	212 (133-231)	153 (115-228)	258 (153-275)	0.80
LDL (mg/dl)	98 (88-178)	133 (113-199)	122 (112-158)	205 (145-233)	0.77
HDL (mg/dl)	43 (38-78)	56 (42-75)	54 (48-82)	73 (58-83)	0.36
Albumin (g/dl)	4.1 ± 1.6	3.2 ± 1.4	3.1 ± 1.7	3.7 ± 1.3	0.85
cIMT (mm)	0.488 ± 0.06	0.425 + 0.12	0.502 ± 0.06	0.506 ± 0.11	0.26
% change in FMD	13 ± 5.3	4.3 ± 13.6	9.8 ± 14	3.8 ± 9.6	0.62
CrPWV (m/s)	6.16 ± 1.6	6.2 ± 1.2	5.38 ± 1.1	7.09 ± 1.32	0.31

There was no significant difference in mean cIMT in common carotid artery between the study and control groups. We did not find any significant difference in absolute change in FMD nor % proportionate change in FMD pre and post-dilatation. Similarly, we could not find any significant difference among the subcategories of NS between cIMT or FMD (Tables 3, 4).

**Table 4:** Comparison of radiological and physiological parameters between study and control group cIMT - carotid Intimal medial thickness; CCA - common carotid artery; FMD - flow-mediated dilatation; SBP - systolic blood pressure; DBP - diastolic blood pressure; crPWV - carotid radial pulse wave velocity; RHI - reactive hyperemia index; AI - augmentation index T test or Wwilcoxon signed rank was applied for analysis.

Parameter	Study group (n=33) Mean ± SD	Control group (n=39) Mean ± SD	p-value
Right CCA (mm)	0.531 ± 0.0.08	0.519 ± 0.0.05	0.46
Left CCA (mm)	0.521 ± 0.0.07	0.514 ± 0.0.04	0.66
Mean cIMT(mm)	0.473 ± 0.0	0.478 ± 0.03	0.90
Z score mean cIMT	0.08 ± 1.20	-0.08 ±0.77	0.50
Absolute change in FMD (mm)	0.12 ± 0.11	0.15 ± 0.13	0.41
% Change in FMD	5.93 ± 6.27	5.53 ± 5.1	0.78
Central SBP (mmHg)	101 ± 8	103 ± 9	0.52
Central DBP (mmHg)	74 ± 9	71 ± 12	0.47
Brachial SBP (mmHg)	105 ± 10	105 ± 7	0.74
Brachial DBP(mmHg)	65 ± 8	63 ± 10	0.34
crPWV (m/s)	5.62 ± 1.76	6.53 ± 2.23	0.1
Reactive hyperemia index (RHI)	1.06 ± 0.11	1.24 ± 0.7	0.30
Augmentation index (AI)	56.4 ± 26.3	70.2 ± 15	0.11

No significant difference was observed in the reactive hyperemia index and carotid to radial pulse wave velocity among the study and control groups. Similarly, central BP and office BP are also comparable in both groups. (Table 4) There was a significant correlation of percentage change in FMD with BMI, total cholesterol (TC), triglycerides (TG), LDL, and the duration of disease. We observed a significant correlation between age with crPWV and RHI (Table 5). There was no significant correlation found between physiological and radiological parameters among the study and control groups (Table 6).

**Table 5:** Correlation between anthropometry, radiological, and physiological parameters Pearson correlation test was applied and the r value was mentioned. FMD - flow-mediated dilatation; cIMT - carotid intima medial thickness; RHI - reactive hyperemia index; AI - augmentation index; crPWV - carotid radial pulse wave velocity; LDL - low-density lipoprotein

Parameters	% change in FMD	cIMT	RHI	AI	crPWV
Age					
Pearson correlation	0.15	0.107	0.26	-0.253	0.38
p-value	0.22	0.4	0.02	0.2	0.002
BMI					
Pearson correlation	-0.374	-0.025	-0.124	-0.034	0.001
p-value	0.002	0.844	0.3	0.86	0.99
Total cholesterol					
Pearson correlation	-0.362	0.047	-0.11	-0.289	0.08
p-value	0.003	0.71	0.32	0.14	0.5
Triglycerides					
Pearson correlation	-0.384	0.035	-0.07	-0.292	0.13
p-value	0.001	0.78	0.56	0.14	0.26
LDL					
Pearson correlation	-0.288	0.003	-0.1	-0.173	0.11
p-value	0.019	0.97	0.4	0.38	0.32
Number of relapses					
Pearson correlation	0.233	-0.006	0.09	0.45	0.19
p-value	0.216	0.97	0.58	0.1	0.27
Duration of disease					
Pearson correlation	0.488	0.121	0.02	-0.212	0.2
p-value	0.006	0.51	0.87	0.46	0.26

**Table 6:** Correlation between radiological and physiological parameters FMD - flow-mediated dilatation; cIMT - carotid intima medial thickness; RHI - reactive hyperemia index; AI - augmentation index; crPWV - carotid radial pulse wave velocity Pearson correlation coefficient was calculated.

Parameters	% change in FMD	cIMT
RHI		
Pearson correlation	0.088	-0.235
p-value	0.48	0.06
AI		
Pearson correlation	0.194	-0.072
p-value	0.34	0.73
crPWV		
Pearson correlation	-0.108	-0.011
p-value	0.386	0.93

## Discussion

NS in children is considered to be a moderate risk factor for cardiovascular morbidity in the long term. The major risk factor associated with cardiovascular morbidity is dyslipidemia and hypertension in these children. In the current study, we did not find any significant difference in the cIMT among children with NS and controls. There was no significant difference in the FMD in the groups, too. 

In our study, 33 children with NS were enrolled. A male preponderance with a male-to-female ratio of 2.3:1 was observed. Dyslipidaemia was seen in more than 50% of children, even during remission. Hypercholesterolemia was documented in 73% of children, whereas hypertriglyceridemia and increased LDL were observed in 79% and 45% of children, respectively. A study by Merouani et al. compared plasma lipid profiles in children with NS at remission and reported that plasma total and LDL-cholesterol levels were above the 95th percentile for age and gender in 48% of patients [16]. A high prevalence of dyslipidemia was also reported in patients with FRNS (hypercholesterolemia 81%) during clinical remission by Tsukahara et al. [17]. Similar to our study, Mahmud et al. also reported higher mean levels of lipids than those of the controls [18]. In contrast, Rahul et al. reported a lower prevalence of dyslipidemia in children with NS [19]. A higher proportion of children had dyslipidemia in our study due to more children in the SDNS and FRNS categories.

Hypertension was seen in almost 36% of our children with NS (27% stage 1 hypertension and 9% stage 2 hypertension) classified as per the recent AAP 2017 recommendation for childhood hypertension. Similar results had been reported by Gheisari et al. (23.4%) and Kontchou et al. (34%) during the remission of disease [20]. Though office BP showed hypertension, we could not find hypertension in central BP monitoring children with NS in our study. We could not find any statistically significance difference between the systolic and diastolic BP between children with NS and the control group. Similar results have been reported by Rahul et al.(19)and Alves et al. [21]. However, Alves et al. reported a significantly higher Z score for systolic BP in children with NS. 

There was no statistically significant difference between mean cIMT in children wth NS as compared to the controls. However, Rahul et al. and Ahmed et al. reported significantly higher mean cIMT in children with NS compared to the controls [19,22]. In our study, there was no significant correlation found between cIMT and duration of disease. This may be due to a smaller sample size and short duration of the disease.

We did not find any significant difference either in absolute change in FMD or % proportionate change in FMD pre and post-dilatation. A study done by Youssef et al. documented less change in brachial artery diameter in children with NS as compared to the control group [23]. In another study, children with NS of age group 1-13 years had a significantly lower proportionate change of FMD (5.65 ± 6.08%) as compared to controls (15.21±9.41%) (19). In the current study, no significant difference was observed in reactive hyperemia index and carotid to radial pulse wave velocity between the study and control groups. Though we have observed lower RHI in children with nephrotic syndrome, which is similar to results observed in children with obesity and T1 DM who are at risk of endothelial dysfunction [24,25], it is statistically not significant. Results of the same cannot be generalized as there is a significant age difference at baseline, which may be a confounding factor. No significant difference was observed among other parameters, such as the augmentation index/central BP, though we had done it in a small number of children. There is only one study that has used PWV and augmentation index to document arterial stiffness in children with NS, as per our knowledge. Alves et al. did not find any significant difference in PWV in children with NS as compared to controls (4.53 ± 0.33 m/s vs 4.52 ± 0.28 m/s, p= 0.919) [22]. 

There was a significant correlation between the percentage change in FMD with BMI, total cholesterol (TC), triglycerides (TG), LDL, and duration of disease. We observed a significant correlation between age and crPWV and RHI. No significant correlation was found between BMI, lipid profile, duration of disease, or the number of relapses with physiological parameters (crPWV, RHI, AI). We observed a positive correlation between PWV and BMI. This finding suggests that though PWV is used as a marker for arterial stiffness, largely influenced by factors like age, height, and body size, which is also observed by Brecheret et al. [26]. The correlation among physiological parameters and AI is difficult to interpret because of the small sample size. There was no significant correlation found between physiological and radiological parameters between the study and control groups. The limitations of the study was not having age-matched controls with the study group due to restricted OPD during the COVID period and hence faced difficulty in enrolling healthy age-matched controls. We were unable to conduct ambulatory blood pressure monitoring (ABPM), which could have potentially overlooked masked hypertension in these children.

## Conclusions

The mean carotid intima-media thickness (cIMT) is similar in both groups. No statistically significant difference is observed in the absolute change in flow-mediated dilation (FMD) or the percentage proportionate change in FMD before and after dilatation in both the study and control groups. Nevertheless, the reactive hyperemia index (RHI) is notably lower in children with NS compared to controls, requiring further validation through additional studies. Consequently, it is crucial to carefully monitor children with NS for endothelial dysfunction and arterial stiffness.
